# Thrombolysis after cardiopulmonary resuscitation in myocardial infarction with abdominal pain as the first presentation: A case report

**DOI:** 10.1097/MD.0000000000029114

**Published:** 2022-04-22

**Authors:** Yang-Jian Zheng, Wen-na Wang, Han-li Lin, Ya-nan Wu

**Affiliations:** a Department of Cardiololy, Putuo Hospital, Zhoushan, Zhejiang Province, China; b Department of Emergency, Putuo Hospital, Zhoushan, Zhejiang Province, China.

**Keywords:** CAG, cardiopulmonary resuscitation, case report, STEMI, thrombolysis

## Abstract

**Rationale:**

Thrombolysis after cardiopulmonary resuscitation in patients with acute ST-segment elevation myocardial infarction (STEMI) is controversial. This case report describes a successful thrombolysis after resuscitation in delayed-diagnosis STEMI.

**Patient concerns:**

A 58-year-old man presented with acute abdominal pain as the first symptom of a subsequent STEMI diagnosis. When he returned to the clinic after having been assisted with abdominal pain relief, he suffered a sudden cardiac arrest. Cardiopulmonary resuscitation was performed immediately, and thrombolysis was carried out for his anterior STEMI. He was successfully resuscitated in a short period of time.

**Diagnosis:**

The patient was diagnosed with acute and extensive anterior STEMI. The D-dimer level was normal, and pericardial effusion was ruled out.

**Interventions:**

After successful resuscitation, the patient received half-dose alteplase thrombolytic therapy. After a few days, the patient was transferred to a general ward. Coronary angiography revealed unobstructed flow in the left anterior descending artery.

**Outcomes:**

The ST segment of the patient gradually declined after thrombolytic therapy, and the myocardial injury marker levels increased. A small amount of pleural fluid in the lungs and pulmonary infection were observed. With effective diuretic, anti-infective, and other treatments, the patient's condition gradually improved, the ventilator was removed, and vasoactive drugs were successfully discontinued. Coronary angiography revealed that the flow of the culprit artery was unobstructed, and a drug-coated balloon was implanted. No wall motion abnormalities were detected on echocardiography, and the patient recovered well.

**Conclusions:**

In patients with abdominal pain as the first presentation, a simple initial electrocardiogram may help reduce the risk of missed STEMI diagnosis. Thrombolysis after successful resuscitation is an effective treatment for these patients. However, the effects of thrombolysis after resuscitation remain unclear. The point of dispute lies in the effectiveness and safety of thrombolysis (primarily for bleeding). Prompt thrombolysis would lead to a better prognosis if spontaneous circulation can be restored within 10 minutes.

## 1 Introduction

Patients with cardiac arrest (CA) can obtain recovery of spontaneous circulation (ROSC) after effective cardiopulmonary resuscitation (CPR). Primary percutaneous coronary intervention (PPCI) is an effective treatment for patients with definite acute ST-segment elevation myocardial infarction (STEMI), regardless of resuscitation duration. However, postresuscitation thrombolytic therapy is controversial, and guidelines do not consistently recommend it because of the risk of bleeding. Li et al^[1]^ demonstrated that thrombolysis had similar efficacy as that of percutaneous coronary invention in patients with return of ROSC after CA. Thrombolysis following ROSC in patients with a delayed diagnosis of STEMI has rarely been reported. This report presents a case of thrombolysis after cardiopulmonary resuscitation for myocardial infarction with abdominal pain as the first presentation.

## 2 Case presentation

A 58-year-old man with a history of hypertension and smoking was admitted to the emergency department on May 15, 2021, by his family after 2 hours of abdominal pain following a dinner drink. The patient presented with persistent severe pain in the middle and upper abdomen, no nausea or vomiting, no fever or cough, and no chest tightness or dyspnea. On examination, the blood pressure was 166/75 mm Hg; the patient was conscious, gave pertinent answers, showed painful appearance; there was no cyanosis of the lips, no puffiness of the face, no pallor of the lid conjunctiva; soft neck with no filling and distension of the jugular veins, coarse breath sounds in both lungs, no obvious dry and wet rales, no large heart borders, no palpable tremor; heart rate of 72 beats/min, regular rhythm, no murmur, and no extra heart sounds. The abdomen was flat and soft, the liver and spleen were not palpable under the ribs, the upper and middle abdomen was markedly painful with no rebound pain, Murphy's sign was negative, there was no pressure pain at McKay's point, there was no percussion pain in the liver or both kidney areas, bowel sounds were slightly active, and there were no abnormal findings in the extremities or neurological examination. Results of the routine blood tests, MB isoenzyme of creatine kinase (CK-MB), troponin, D-dimer, blood amylase, and computed tomography of the whole abdomen and chest were normal, and no electrocardiogram (ECG) was obtained on admission. Therefore, an initial diagnosis of acute gastritis was established. Omeprazole therapy, 654-2 injection, and appropriate rehydration treatment were prescribed, and the patient had gradual abdominal pain relief. However, the patient had sudden loss of consciousness during the follow-up between visits. He did not respond to the calls; there was loss of carotid pulsation, and unheard heart sounds. Initial CPR was immediately performed, the defibrillator showed ventricular fibrillation, and the patient had sinus rhythm and restoration of autonomic circulation after multiple defibrillations, but the patient remained unconscious.

**The following timeline describes the changes in the patient's condition at various time points and changes in the electrocardiographic findings after thrombolytic therapy and thrombolysis.**

At 22:44 pm, The patient returned to the clinic with significant relief of abdominal pain but suddenly became unconscious with no response to calls, and no aortic pulses were noted. CPR was performed immediately, and the patient was placed in the resuscitation room for further resuscitation.

At 22:45 pm, ECG monitored ventricular fibrillation, and asynchronous 150-J defibrillation was performed for intermittent ventricular fibrillation, up to 150 mg of amiodarone, IV push, continuous chest compressions, and respiratory support.

At 22:48 pm, ECG monitored ventricular fibrillation, asynchronous 150-J defibrillation was performed, ventricular tachycardia was noted, and amiodarone 150 mg, IV push, continued with 450 mg amiodarone 10 mL/h, and micropump IV push was administered.

**Figure 1:**
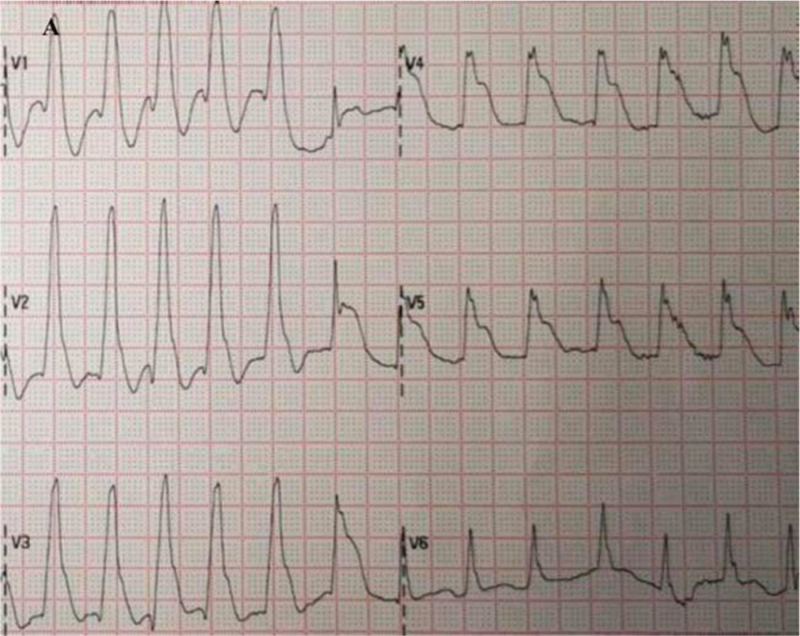
First ECG after resuscitation: atrial fibrillation rhythm with elevated R-wave amplitude in the anterior wall leads, giant R-wave manifestation. ECG = electrocardiogram.

At 22:56 pm, the first ECG after resuscitation showed that the R-wave amplitude in leads V1 to V3 was significantly elevated, the R-wave in leads V2 to V5 was large, and the QRS in leads V3 to V5 was widened (Fig. 1). The infarction was injected intranasally with a packet of drugs, and acute amiodarone was pumped in, while the family was contacted by phone urgently for emergency PCI, but they refused.

**Figure 2:**
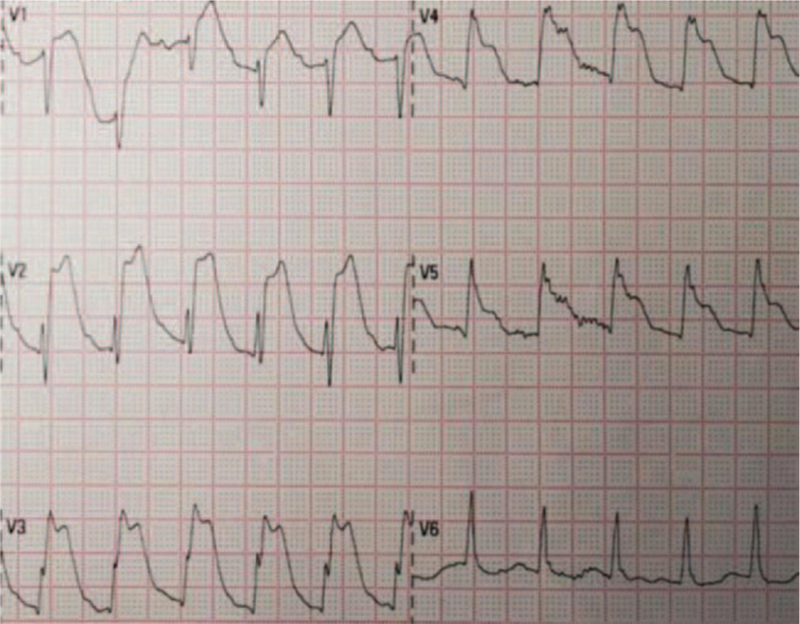
Prethrombolysis ECG after successful resuscitation: decreased R amplitude in anterior wall leads V1 to V3 and evolution toward ST-segment elevation. ECG = electrocardiogram.

Seven minutes later, ECG showed sinus rhythm and a giant “R” with ST-segment elevation in extensive anterior wall leads. The family was asked to agree to perform thrombolytic therapy after removing the fluid from the chest and pericardium. A dose of 42 mg was added to saline solution for 30 minutes (Fig. 2).

**Figure 3:**
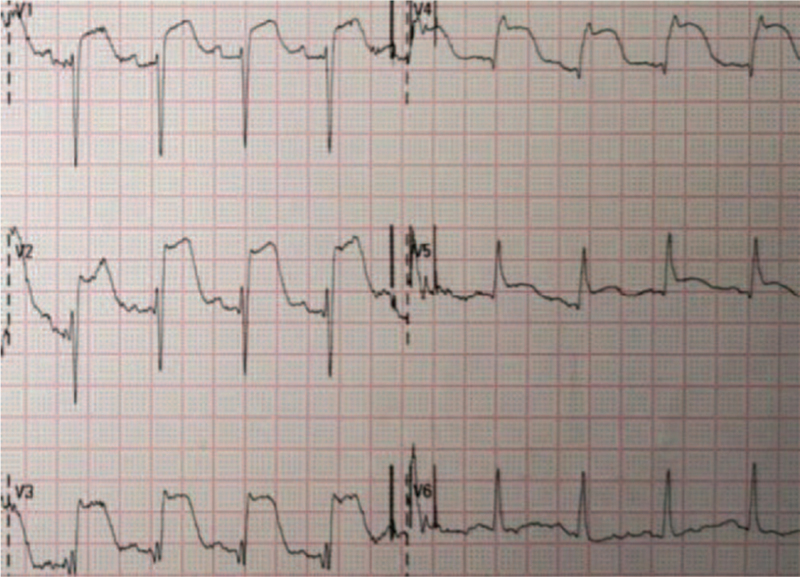
ECG after alteplase administration: sinus rhythm with ST segment elevation in leads V1 to V4, “tombstone-like.” ECG = electrocardiogram.

ECG after alteplase administration showed sinus rhythm with ST segment elevation in leads V1 to V4, “tombstone-like” (Fig. 3).

**Figure 4:**
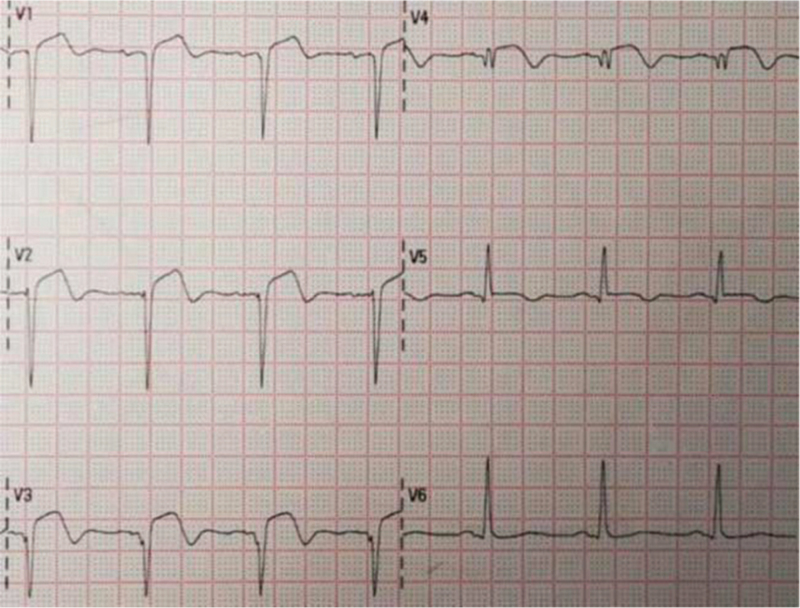
At 2 hours after thrombolysis: sinus rhythm with poor R-wave progression in leads V1 to V5, ST-segment regression, and T-wave positive and negative in both directions.

At 2 hours after thrombolysis ECG showed sinus rhythm (75 beats/min), poor R-wave progression in leads V1 to V5, ST-segment regression, and positive and negative T-waves in both directions > 2 hours after alteplase administration (Fig. 4).

The patient was unconscious throughout the resuscitation and thrombolysis periods and was intubated and ventilated with ventilator-assisted ventilation, maintained with antihypertensive drugs, sedated, and nasally fed. During the intensive care unit stay, the patient could not be effectively deconditioned and developed pulmonary infection and multiple organ impairment. The patient refused PCI treatment and was treated with medications, including low molecular heparin calcium injection 5000 IU subcutaneously every 12 hours, tegretol tablets 90 mg bid, aspirin enteric solution tablet 100 mg bid, Lipitor 20 mg qd, metoprolol extended-release tablet 11.375 mg qd, and intragastric tube injection.

**Figure 5:**
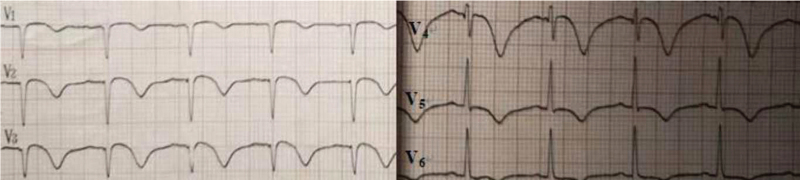
At 35 hours after thrombolysis: sinus rhythm T-wave inversion in leads V1 to V6 and QS pattern in leads V1 to V3.

At 35 hours after thrombolysis, repeat ECG sinus rhythm T-wave inversion in the inferior wall leads, T-wave inversion in leads V1 to V6, QS pattern in leads V1 to V3, and a prolonged QT interval were observed (Fig. 5).

**Figure 6:**
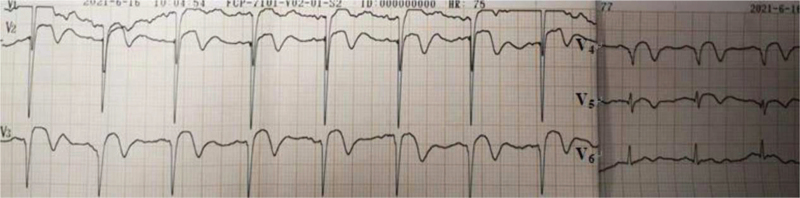
At 7 days after thrombolysis: sinus rhythm with T-wave in leads V1to V5 positive and negative bidirectional and QS pattern in leads V1 to V4.

At 7 days after thrombolysis, the patient was successfully deconditioned and transferred to the general cardiology ward. The results suggested that the sinus rhythm T-wave inversion in the inferior wall leads became shallow, the electrical axis was left-deviated, the T-wave in leads V1 to V5 was positive and negative in both directions, and the QS pattern was noted in leads V1 to V4 (Fig. 6).

During the whole thrombolysis process, the patient had no bleeding complications.

**Figure 7:**
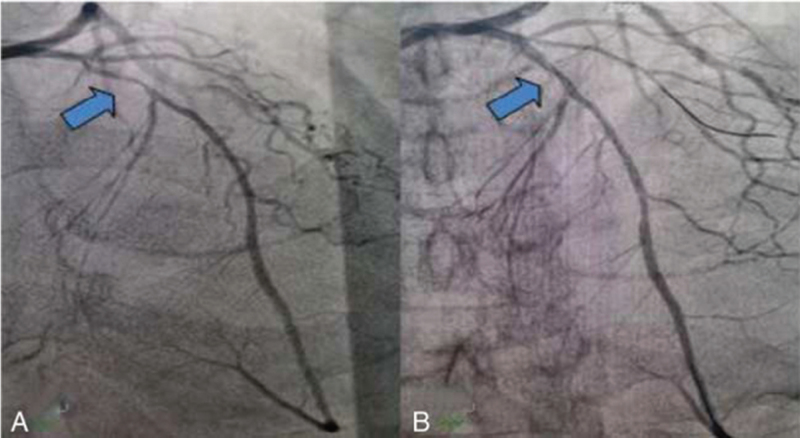
Coronary angiography showing TIMI flow grade 3 with diffuse lesions (up to 90% stenosis) at proximal LAD as indicated by the arrow (A); stenosis disappears after PTCA with a drug-coated balloon (B). LAD = left anterior descending, PTCA = percutaneous transluminal coronary angioplasty, TIMI = thrombolysis in myocardial Infarction.

After obtaining consent from the patient's family, the patient was transferred to the general ward for coronary angiography (Fig. 7).

Cardiac ultrasonography suggested uncoordinated left ventricular wall motion with reduced septal and apical ventricular wall motion, with an ejection fraction of 48%. Chest computed tomography revealed a small pleural effusion. CK-MB and troponin levels after thrombolysis are shown in Table 1. After a series of careful treatments, the patient recovered well, the pleural effusion disappeared, and the patient's general condition was acceptable with good cardiac function at telephone follow-up.

**Table 1:**
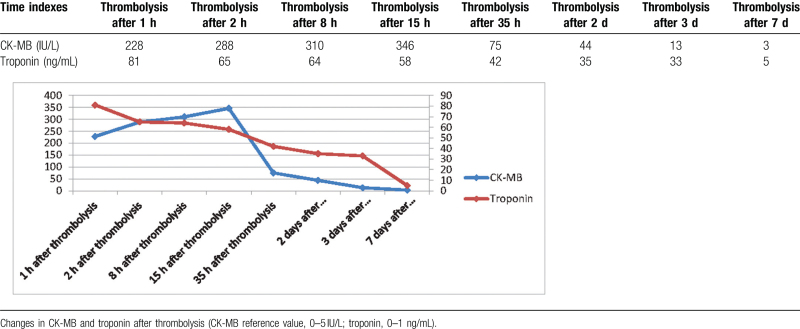
Changes in markers of myocardial injury after thrombolysis.

## 3 Discussion

Acute myocardial infarction is usually manifested as chest pain, chest tightness, or chest discomfort with or without radiation to other parts, as well as dyspnea, sweating, and other typical symptoms, which are relatively easy to identify by clinicians or patients themselves. “Abdominal pain” is a rare first symptom of acute myocardial infarction in acute coronary syndrome, which is an atypical manifestation, and the first symptom is manifested. A meta-analysis^[2]^ showed that atypical presentations in patients with acute myocardial infarction are relatively common in the elderly population and women. Delayed diagnosis and management clearly increase mortality in patients with acute myocardial infarction. In the present case, the patient was admitted with mid-upper abdominal pain, who had a clear history of alcohol consumption and clear abdominal signs (mid-upper abdominal pressure pain), but suddenly gained consciousness when abdominal pain was significantly relieved, and the final diagnosis was anterior STEMI after cardiopulmonary resuscitation. Therefore, it is necessary to be alert for acute coronary syndrome in patients with abdominal pain, even in those with clear causative factors and signs. Bedside ECG is required as early as possible to avoid delays in timely diagnosis and management.

PPCI is the most effective treatment for STEMI, particularly in patients with contraindications to thrombolysis. Thrombolysis within 3 hours of STEMI onset is equivalent to emergency PCI, and current guidelines recommend thrombolysis within 6 hours without PCI or FMC-B > 120 minutes, but there are no current guidelines for thrombolysis after cardiopulmonary resuscitation after CA. Multiple studies on postresuscitation thrombolytic therapy can effectively improve patient prognosis.^[3,4]^As early as 2001, Böttiger et al^[4]^ published a prospective study in The Lancet, which included 90 patients who did not have return of autonomic circulation after 15 minutes of CPR, 68% (27) of the 40 patients who were given rt-PA regained the loop autonomously, and 58% (23) were successfully transferred to the intensive care unit, compared with those without thrombolysis (44% and 30%, *P* < .05). The study by Böttiger et al^[5]^ published in the New England Journal of Medicine focused on thrombolysis, with alteplase thrombolysis, at the time of out-of-hospital cardiac resuscitation, with 525 cases each in the thrombolysis group (74.8% considering myocardial infarction) and the non-thrombolysis group (68.5% considering myocardial infarction). No significant benefit was found in the thrombolysis group in terms of 24-hours and 30-day mortality, autonomic circulation, neurological function evaluation, and survival to patient discharge, but the authors concluded that patients with CA witnessed in this study do not necessarily have myocardial infarction. However, Richling et al ^[6]^ found no difference in neurologic recovery and mortality when they compared thrombolysis with PCI in patients with CA due to STEMI. Hemodynamic studies have indicated that thrombolysis can decrease microthrombosis, improve microcirculation metabolism, and promote neurological recovery.^[7,8]^ Additionally, 2 studies were published in the Chinese Journal of Emergency Medicine in 2008 and 2011,^[9,10]^ with the former including patients who had sudden cardiac death, divided into standard resuscitation combination and thrombolytic resuscitation group. Patients who still did not have return of autonomic circulation within 15 minutes after CPR received urokinase or rt-PA thrombolytic therapy, and the recovery discharge rate in the thrombolytic resuscitation group was significantly higher than the standard resuscitation group (*P* < .05). Bleeding was more frequently noted in the thrombolytic resuscitation group than the standard resuscitation group, but there was no statistical difference, such as skin petechiae, gastrointestinal bleeding, and hemoptysis, and no serious life-threatening bleeding was observed. Similarly, Spöhr et al ^[11]^ noted that thrombolysis resuscitation in patients with acute infarction did not increase the risk of additional bleeding and suggested that thrombolysis should not be a contraindication for such patients. A meta-analysis of the effect and safety of thrombolysis during resuscitation was performed by Li, which included 9 clinical studies, with 2 prospective, randomized controlled and double-blind and 2 prospective, non-randomized controlled studies of which 847 cases were classified in the thrombolysis group and 1028 cases in the nonthrombolysis group. Patients in the thrombolytic resuscitation group had higher survival and discharge rates at 24 hours than those in the nonthrombolytic group (*P* = .02, *P* = .03, respectively). In terms of post-thrombolytic bleeding, the thrombolytic group had a higher incidence of severe bleeding than the nonthrombolytic group (10.6% vs 5.81%, *P* < .005), and the thrombolytic group did not show better neurological function in successfully resuscitated patients than in the nonthrombolytic group (*P* = .35). However, the authors in their discussion analyzed that we cannot ignore the time of CA of the patient in terms of neurological recovery; it does not improve the prognosis even if is effective. To reduce the risk of bleeding complications from thrombolysis after resuscitation, this patient was administered half the dose of alteplase for thrombolysis. Pu et al ^[12]^ found that a half dose of alteplase resulted in good perfusion of the epicardium and myocardium in patients with STEMI within 6 hours of onset if pre-estimated delayed PCI was possible and there is no difference in death, recurrent myocardial infarction, or bleeding compared with the PPCI group.

Arntz et al ^[1]^ retrospectively analyzed the 3-year data of prehospital CPR thrombolysis cases in Berlin, Germany, and concluded that very good survival and neurological outcomes were obtained with selective posthospital resuscitation thrombolysis in patients with highly suspected STEMI. In this study, thrombolytic therapy was initiated by prehospital CPR followed by 12-lead ECG diagnosis (ST segment elevation or newly complete left bundle branch block) and return of autonomic circulation shortly after timely cardiopulmonary resuscitation. They found significantly higher thrombolytic survival rates in patients with autonomic circulation within 10 minutes of CPR.

In this case, CPR was started immediately after CA, and defibrillation was performed several times because CA occurred in the hospital, and the time of circulation recovery was < 8 minutes. The total time from onset, infusion, CPR, and STEMI confirmation to thrombolysis initiation was approximately 5 hours, including phone time with his family member and bedside ultrasonography (pericardial and aortic ultrasound). Two hours of postresuscitation alteplase thrombolysis with ST-segment regression and CK-MB peak in advance indicated that the thrombolytic effect was prospective. Final coronary angiography further confirmed that the flow in the left anterior descending branch was patent, and no thrombus or slow flow was observed. Norepinephrine injection was maintained and withdrawn for 2 days, and oxygenation was good with ventilator support.

ECG of the patient at resuscitation was suggestive of a giant R-wave ECG (Fig. 1), which strongly indicated danger and was predictive of high mortality, as evidenced by the multiple episodes of ventricular fibrillation and ventricular tachycardia that followed. Several ECGs also suggested significant ischemic changes in the anterior and inferior wall ECGs, and the later coronary angiography also indicated that the proximal segment of the left anterior descending branch was then occluded, affecting the innervation of the extensive anterior wall and part supply of the inferior myocardial wall. Certainly, in response to the first postresuscitation ECG, our emergency physicians were still alert to this ECG and recommended early PCI as the best choice; however, the family refused PCI. We performed half-dose alteplase thrombolysis, which achieved good results in combination with ECG and coronary angiography results, and the ECG changes before and after thrombolysis are documented in detail in this report. No bleeding was observed during or after thrombolysis, and the patient's neurological function recovered well. Coronary intervention was performed 3 to 24 hours after thrombolysis according to the guideline recommendations. Although the family initially refused remedial PCI until the patient was transferred out of the general ward and the family agreed to the procedure, the patient eventually achieved a good clinical outcome.

## Author contributions

**Data curation:** Ya-nan Wu.

**Formal analysis:** Yang-Jian Zheng.

**Investigation:** Yang-Jian Zheng, Wen-na Wang.

**Methodology:** Yang-Jian Zheng.

**Project administration:** Han-li Lin.

**Resources:** Yang-Jian Zheng, Han-li Lin.

**Supervision:** Wen-na Wang.

**Validation:** Ya-nan Wu.

**Writing – original draft:** Yang-Jian Zheng.

**Writing – review & editing:** Han-li Lin.
